# Auditory Temporal Processing Abilities in Early Azari-Persian Bilinguals

**Published:** 2013-10

**Authors:** Roya Sanayi, Ghassem Mohamadkhani, Akram Pourbakht, Leila Jalilvand, Shohreh Jalayi, Soudabeh Shokri

**Affiliations:** 1 Department of Audiology, Faculty of rehabilitation, Tehran University of Medical Sciences, Tehran, Iran.; 2 Department of Audiology, Faculty of rehabilitation, Shahid Beheshti University of Medical Sciences, Tehran, Iran.; 3 Department of Physiotherapy, Faculty of rehabilitation , Tehran University of Medical Sciences, Tehran, Iran.

**Keywords:** Auditory perception, Multilingualism, Pitch perception

## Abstract

**Introduction:**

Auditory temporal resolution and auditory temporal ordering are two major components of the auditory temporal processing abilities that contribute to speech perception and language development. Auditory temporal resolution and auditory temporal ordering can be evaluated by gap-in-noise (GIN) and pitch-pattern-sequence (PPS) tests, respectively. In this survey, the effect of bilingualism as a potential confounding factor on auditory temporal processing abilities was investigated in early Azari-Persian bilinguals.

**Materials and Methods:**

In this cross-sectional non-interventional study, GIN and PPS tests were performed on 24 (12 men and 12 women) early Azari-Persian bilingual persons and 24 (12 men and 12 women) Persian monolingual subjects in the age range of 18–30 years, with a mean age of 24.57 years in bilingual and 24.68 years in monolingual subjects. Data were analyzed with t-test using SPSS software version 16.

**Results:**

There was no statistically significant difference between mean gap threshold and mean percentages of the correct response of the GIN test and average percentage of correct responses in the PPS test between early Azari-Persian bilinguals and Persian monolinguals (P≥0.05).

**Conclusion:**

According to the findings of this study, bilingualism did not have notable effect on auditory temporal processing abilities.

## Introduction

In the 21st Century, learning a second language is increasingly important. It is thought that 24% of Iranian people are Azari-Persian bilinguals. The Azari language has a different phonetic, syntactic, and grammatical structure in comparison to the Persian language (1).

Auditory temporal processing is one of the most important capabilities for hearing and speech perception (2). Auditory temporal processing could be the groundwork for many processing abilities such as processing acoustic verbal and non-verbal signals, music perception, rhythm, pitch differentiation, duration, and phonemes (3). Temporal acuity is the ability of apperception of stimulus variations during a given period, such as the demodulation a gap among two sounds or identification of amplitude modulations along a permanent sound (4). Temporal resolution proves to be necessary for speech perception, because provision of information relating to vowels, consonants, syllables, and expression boundaries is incumbent for discernment of speech and language evolution (5). The gap-in-noise (GIN) test developed by Musiek et al (2005) investigates auditory temporal resolution (6). Auditory temporal ordering is another sub-set of auditory temporal processing for perception of the authentic subsequence of sounds. The pitch-pattern-sequence (PPS) test developed by Musiek et al(1994) investigates auditory temporal ordering. These tests are appropriate for children and adults with different languages because of their non-verbal nature (2). 

Some studies have shown that bilinguals have a poorer speech perception in noise in comparison to monolinguals (7–9). In contrast, others revealed that bilinguals rapidly detect lingual alterations during speech and that they can also consciously manage lingual variations (10). Therefore, this study aimed to evaluate auditory temporal resolution and auditory temporal ordering in early Azari-Persian bilinguals in order to determine that exposure to a second language facilitates central auditory processing or that interference of the information from two languages causes auditory processing obstacles.

## Materials and Methods

This cross-sectional non-interventional study was performed in the Audiology clinic of the Rehabilitation Faculty of Tehran University of Medical Sciences in 24 Azari-Persian bilingual subjects (12 men and 12 women) and 24 Persian monolingual individuals (12 men and 12 women), with a mean age of 24.57±2.6 years (bilingual subjects) and 24.68±2.71 years (monolingual subjects). The bilingual group consisted of early bilinguals who attained the two languages before the age of 6 years (10). Auditory temporal processing skills at age 11 are developed and are little influenced by learning (11). Inclusion criteria included age range between 18 and 30 years, normal hearing in audiometry tests (bilateral hearing thresholds ≤20 dB HL) (12), bilateral type An-tympanogram and normal acoustic reflexes in both ears, right handedness (13). Exclusion criteria included previous history of ear disease, head injury, brain surgery, background of epilepsy and any consumption of psychotherapeutic drugs or musical experience. All subjects were informed about the nature and purpose of the study before consenting to participate. Pure-tone audiometry was performed using an AC 40 audiometer (Interacoustics, Denmark) in a sound-proof booth and earphones TDH-39. Tympanometry and acoustic reflex tests were accomplished using a Zodiac 90 1tympano- meter (Madsen, Denmark).

GIN and PPS tests were conducted in a sound-treated room. Stimuli were presented via a CD player through audiometer (AC40) and TDH-39 earphones. Stimuli of GIN are a set of bursts of white noise with a6-s duration up to three gaps in every segment. Gap durations were 2–3–4–6–8–10–12–15 and 20 ms and the silence epoch between every set was 6 s. The test was administered at 50 dB SL compared with the speech reception threshold. An approximate gap threshold and percentage of the correct responses are two criteria for this test (14). Furthermore, the PPS test consists of 60 items. Each item is a set of three pure tones with two different pitches, with a low-frequency tone at880 Hz and a high-frequency tone at 1122 Hz. The duration of every tone is 200 ms with 10-ms rise and fall time. These tones are separated by 150-ms intervals and the silence epoch between every set is 6 s (15). Stimuli were presented at 55 dB SL compared with the threshold of 1000 Hz. The percentage of correct responses is the outcome for this test. Subjects were instructed to listen carefully to the practice part of the GIN and PPS tests and then to answer the test items. 

Data were analyzed using a t-test because the distribution of data was normal according to the Kolmogrov-Smirnov test, and p<0.05 was statistically considered significant. SPSS software version 16 was used in this study.

## Results

**Fig 1:**
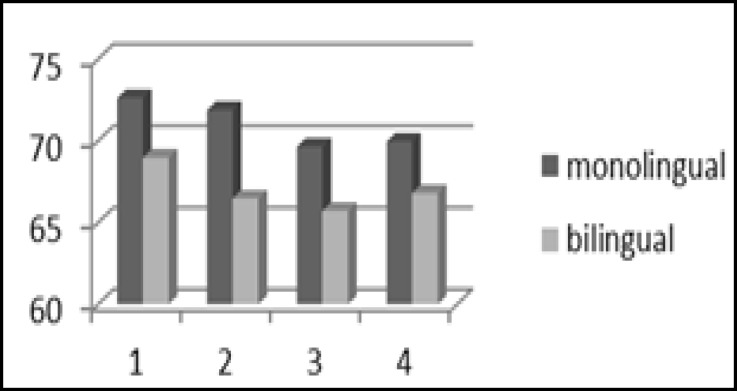
Percentage of correct responses in the GIN test in monolinguals and bilinguals

**Fig2:**
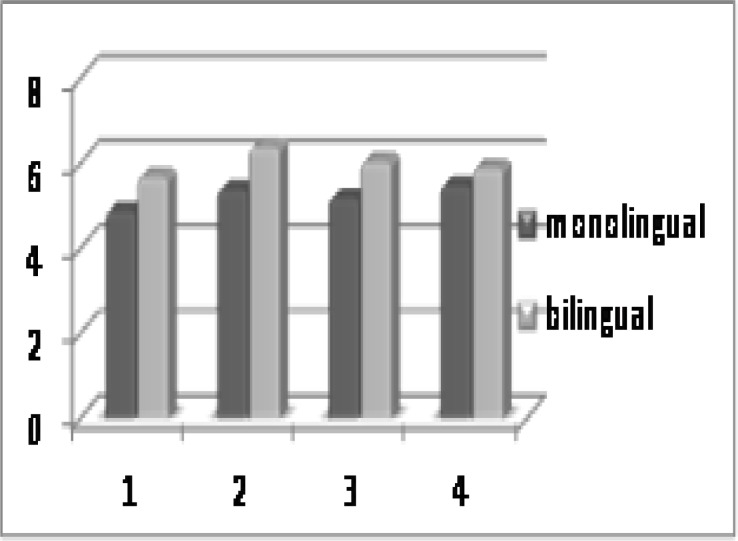
Mean approximate gap thresholds of GIN test in bilinguals and monolinguals

The mean percentage of correct responses and mean approximate gap thresholds for the GIN test are shown in Figures 1 and 2, respectively. Figure 1 shows that in every four lists of the GIN test, the mean percentage of correct responses was slightly poorer in bilinguals than monolinguals, but there was no significant difference between groups (P>0.05). Also there was no significant difference in mean approximate gap thresholds of the GIN test between groups (P>0.05).

**Fig 3:**
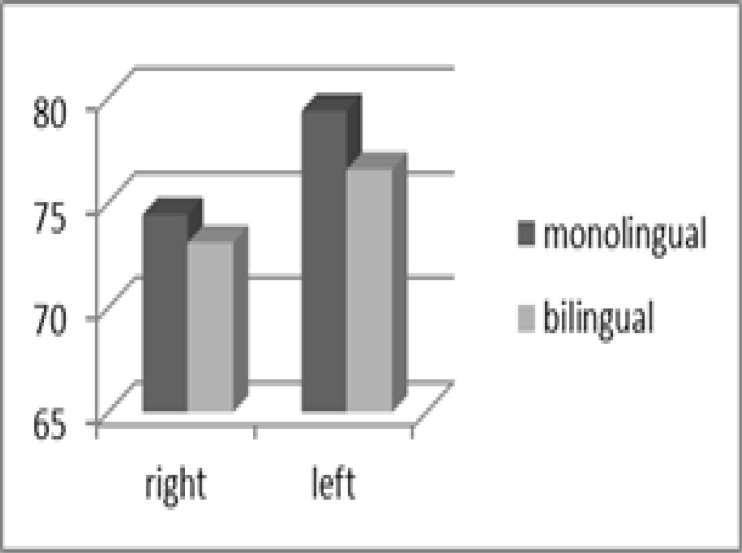
Mean percentage of correct responses in the PPS test in bilinguals and monolinguals

Figure 3 illustrates the mean percentage of correct responses in the PPS test showing a slightly improved result in monolinguals compared with bilinguals but no statistically significant difference between groups (P>0.05). 

The mean percentage of correct responses and mean approximate gap thresholds in the GIN test and mean percentage of correct responses in the PPS test in the right and left ears of the two sexes are depicted in Table 1. Statistical analysis using an independent t-test showed there were no significant differences in mean scores of GIN and PPS parameters between the right and left ears among men and women in the entire study population (P>0.05). 

**Table 1:** Descriptive statistical analysis of GIN and PPS parameters in the study population (n=40).

**Sex/Ear**	**Women /right** $\overset{\text{̅}}{X}$ ** (SD)**	**Women/left** $\overset{\text{̅}}{X}$ ** (SD)**	**Men/right** $\overset{\text{̅}}{X}$ ** (SD)**	**Men/left** $\overset{\text{̅}}{X}$ ** (SD)**
Mean correct responses of GIN	68.64±13.31	69.265±11.77	69.836±9.99	68.3±13.009
Mean of gap threshold in GIN	5.52±1.92	5.8±2.01	5.375±1.29	5.825±2.42
Correct responses of PPS	68.425±24.61	73.755±21.46	79.13±15.136	82.25±13.19
				

## Discussion

It is known that the encoding of auditory temporal sound information establishes substantial information relating to the nervous establishment. For speech perception, the listener should analyze the acoustic cues that speaker has produced.

In this study there was no significant difference between mean percentage of correct responses in the GIN test between Azari-Persian bilinguals and Persian monolinguals. Other studies that applied words as the materials demonstrated that bilinguals have poorer scores in word recognition in noise in comparison to monolinguals (7-9). The stimulus in these studies was words, while in our study, the stimulus was a burst of noise with intervals that should be distinguished. Hence it appears that our stimuli were not sufficiently challenging enough in comparison to words in the evaluation of the central auditory system. The GIN test is perhaps a reflection of the identification of diminutive phonetic components in discourse. Although the GIN test exerts non-verbal segments, the modus in which these stimuli are processed by speakers of different languages may vary because of the specific phonetic aspects of each language (6,16–17). As the Azari language has a different phonetic, syntactic, and grammatical structure in comparison to the Persian language, slight differences in the results could be accounted for by discrepancies between the two languages. The auditory temporal ability for processing basic aspects of sounds is substantial. If a person cannot recognize the time pattern they cannot extract and use prosodic features of speech such as rhythm, stress, and tone. This means the listener cannot identify key words in a sentence and is not able to percept the emphasis. Such individuals may be incapable of discriminating subtle differences in meaning based on changes in stress or tone alone (11). 

The lack of an ear advantage observed in the GIN test is consistent with previous studies (6,16–18). The lack of difference between the ears illustrates that auditory temporal resolution ability is similar in both ears (18).

Furthermore, there were no statically significant principal discrepancies in the results of the mean gap thresholds and mean percentage of correct responses in the GIN test between men and women. Earlier studies (16) have demonstrated a slight discrepancy in the data among two sexes, with men achieving the better results (17–11,19). However, like the study of Balen et al (20), this criterion was not obvious in present study.

Other capability that was investigated in the current study was auditory temporal ordering via the PPS test. Pitch is one of the language's most information-rich segments. Use of tone in language is beneficial for the evaluation of linguistic usage. Overall pitch may be a reflection of the fluctuations in pitch patterns at a syllable level that may be lexically relevant.

Our study also highlights that auditory temporal ordering was not distinct in Azari-Persian bilinguals and is also considerable in Persian monolinguals. A basic ability for effectuating this test is frequency discrimination. There is growing evidence to demonstrate that the neural representation of pitch may be affected by one's experience with language or music at the subcortical as well as at the cortical levels of processing. According to the study of Krishnan and colleagues examining frequency following response, pitch tracking in tonal languages (Thai, Mandarin) was superior to that in a non-tonal language (English) (21). Also Onoda et al illustrated that auditory experience that has been created by music or Japanese language facilitates frequency pattern recognition (22). From invariant results in Azari-Persian bilinguals compared with monolinguals, several probabilities could be inferred. First, the learning of a second language means no more language experience in Azari-Persian bilinguals. Second, language experience may not be sufficiently great that it affects the behavioral results of the PPS test; while electrophysiological procedures that are more precise than the behavioral tests can illustrate this ability. Third, it is likely that the language experience in Azari is not sufficiently challenging, like a musical experience, that it can influence pitch discrimination. In a survey by Gregory (1982), it was shown that the 75% of subjects had a left-ear advantage in pitch discrimination, whereas 20% had a pitch-discrimination preference in the right ear (23). In the present study, monolingual and bilingual subjects had priority in the left ear, although this was not statically significant. Moreover, this finding could be clinically relevant. Furthermore, our data illustrated a marginal increased performance in men than women, whereas Onoda et al (2006) found no difference between men and women. Rosen (1991) demonstrated that high testosterone levels can provoke the development of the right hemisphere and can conversely delay the development of the left hemisphere (24). Consequently, we can conclude that the development of men over women in temporal processing tasks may be influenced by a hormonal effect.

## Conclusions

Over all, it can be concluded from this study that the auditory experience through the Azari language has no prominent effect on auditory temporal resolution or auditory temporal ordering processing. Slight differences between GIN and PPS test parameters could be ascribed to discrepancies between the two languages; in other words, the different phonetic, syntactic, and grammatical structure of the Azari language compared with the Persian language. 
